# Specificity of executive function and theory of mind performance in relation to attention-deficit/hyperactivity symptoms in autism spectrum disorders

**DOI:** 10.1186/s13229-017-0177-1

**Published:** 2017-11-09

**Authors:** Steve Lukito, Catherine R. G. Jones, Andrew Pickles, Gillian Baird, Francesca Happé, Tony Charman, Emily Simonoff

**Affiliations:** 1 Department of Child and Adolescent Psychiatry, Institute of Psychiatry, Psychology and Neuroscience, King’s College London, London, UK; 2 School of Psychology, Cardiff University, Cardiff, UK; 3 Department of Biostatistics and Biomedical Research Centre for Mental Health, Institute of Psychiatry, Psychology and Neuroscience, King’s College London, London, UK; 4 Guy’s and St Thomas’ NHS Foundation Trust, King’s Health Partners, London, UK; 5 Institute of Psychiatry, Social, Genetic and Developmental Psychiatry (SGDP) Centre, Psychology and Neuroscience, King’s College London, London, UK; 6 Department of Psychology, Institute of Psychiatry, Psychology and Neuroscience, King’s College London, London, UK

**Keywords:** Autism spectrum disorder, Attention-deficit/hyperactivity disorder, Comorbidity, Executive function, Theory of mind, SNAP

## Abstract

**Background:**

Individuals with autism spectrum disorder (ASD) frequently demonstrate symptoms of attention-deficit/hyperactivity disorder (ADHD). Previous findings in children with ASD have suggested that these symptoms are associated with an impairment in executive function (EF) abilities. However, studies rarely considered this association within a single framework that controls for other related factors such as Theory of Mind (ToM) abilities and ASD symptoms.

**Methods:**

We used structural equation modeling to explore the relations among EF, ToM, and symptoms of ASD and ADHD, using data from a population-based sample of 100 adolescents with ASD and full-scale IQ ≥ 50 (the Special Needs and Autism Project (SNAP) cohort). The study used a multi-measure and multi-informant approach, where performance of inhibition, planning, switching, and working memory tasks indexed EF and performance on tasks involving mentalizing indexed ToM. Measures of ASD and ADHD symptoms included parent and teacher reports and direct observation of the children. Shared source of symptom reporting was accounted for with a parental rating latent factor indexed by symptom measures reported by parents.

**Results:**

Impairments in EF abilities were specifically associated with ADHD symptoms while impaired ToM was specifically associated with ASD symptoms, when accounting for the associations of each cognitive domain with the other factors. ASD and ADHD symptom latent factors were also correlated, but this association became nonsignificant once the shared source of reporting from parents was accounted for and within a model that also controlled for the correlated pathway between EF and ToM factors. The specific relations between the cognitive domains and behavioral symptoms remained even after controlling for IQ.

**Conclusions:**

In this ASD sample, symptoms of ADHD and ASD are underpinned by separate cognitive domains. The association between EF and ToM impairments is a likely partial explanation for the co-occurrence of ADHD symptoms in ASD, but the role of shared reporting effects is also important and supports the inclusion of independent informants and objective measures in future research.

**Electronic supplementary material:**

The online version of this article (10.1186/s13229-017-0177-1) contains supplementary material, which is available to authorized users.

## Background

Autism spectrum disorder (ASD) is a condition affecting 1–2% children worldwide [1]. Individuals with ASD are impaired in reciprocal social communication and interaction and display various stereotyped and repetitive behaviors [2]. Many children with ASD also meet the criteria for attention-deficit/hyperactivity disorder (ADHD), with rates of approximately 30–60% in community samples [3–5] compared to 5–7% for ADHD in the general population [6]. Both ASD and ADHD are associated with highly overlapping cognitive impairments (e.g., [7–9]). Therefore, neurocognitive approaches can be useful for explaining the mechanisms underpinning the co-occurring ADHD in the ASD population [10–12].

Two disorders can co-occur beyond chance level due to artifactual or non-artifactual reasons [13, 14]. In the clinical setting, the co-occurrence of two disorders can arise due to sampling or ascertainment biases, resulting in artifactual increase of its prevalence. However, these mechanisms cannot be the sole explanation for the co-occurrence of ASD and ADHD because their high rates have been observed in population-based and epidemiological samples [3–5]. Indeed, several models of non-artifactual comorbidity of ASD and ADHD have been proposed recently [10–12, 15]. The “additivity model” is one such model [11]. The model hypothesizes that the co-occurrence of ASD and ADHD arises from separate but correlated risk factors or liabilities and results in the “additive combination of two separate nosologies” [14]. To investigate this model, neurocognitive studies typically use group comparison or factorial design, contrasting individuals with pure ASD or ADHD, combined ASD + ADHD, against those with typical development. In these studies, the pure ASD or ADHD groups are expected to demonstrate unique cognitive profiles, of which combination characterizes the cognitive performance of the ASD + ADHD group. The model thus predicts a double dissociation between the cognitive correlates of ASD and ADHD traits [14].

In the context of cognitive function, impairments of theory of mind (ToM) and executive function (EF) cognitive domains are often reported in children with ASD (e.g., [9, 16–19]). Central to ToM is the ability to mentalize, that is, to attribute mental states such as beliefs, desires, feelings, and intentions of others. Impaired ToM abilities are thought to be developmentally specific to ASD [20, 21] and have been reported to much lesser extent in ADHD (e.g., [9, 22, 23]). Often reported in both disorders are EF impairments, which are usually inferred from performance of inhibition, working memory, cognitive flexibility, and planning tasks (see e.g., [24, 25]). The impairments of EF have been reported especially in ADHD [26–28], and it is uncertain presently if executive dysfunctions truly characterize ASD. Findings of impairments across studies have been heterogeneous (e.g., [17, 29, 30]), and the performance of the EF tasks are rarely correlated with ASD symptom severity, except for between cognitive flexibility deficits and repetitive behavior (e.g., [31–34]). Evidence also suggests that EF impairments in the ASD population are associated with co-occurring ADHD traits. For instance, Corbett et al. [35] found that the inhibitory impairments among ASD children fell to trend level after excluding those with additional ADHD, while Buehler et al. [36] showed increasing motor inhibition impairments in the ASD + ADHD relative to the pure ASD group. Others have also reported increased impairments of sustained attention and working memory in the combined vs. the pure ASD group [11, 12, 15, 37, 38].

Those findings suggest dissociable relations between EF and ToM impairments and the ADHD or ASD traits among individuals with ASD [39], but there are limitations to this interpretation. Firstly, the pattern of increased executive dysfunctions in the combined relative to the pure ASD group have not been observed in *every* EF subdomain (e.g., in the domains of planning and cognitive flexibility [11, 40, 41]), although presumably, these subdomains have specific characteristics beyond the “common” EF [25, 42] that are unrelated to the ADHD traits. Furthermore, most studies tend to use single measures for defining symptoms of ASD and ADHD, completed typically by a single informant (e.g., parents for pediatric studies). Such approach may increase the magnitude of associations between symptom domains because of the shared source of information, in this case between, ASD and ADHD symptoms [39]. Finally, most studies have tested the links between each domain of cognition (ToM or EF) and behavioral traits in separate studies [39]. Thus, possible influences each factor might have on the relation between the other cognitive domain and behavioral traits might be obscured. Moderating influences could exert for instance through pathways between EF and ToM (e.g., [43–46]) or between the symptom domains of ASD and ADHD (e.g., [47–49]), which should be controlled within one single framework.

We explore the associations among EF, ToM, and symptom domains of ASD and ADHD and address the above challenges in the present study. In line with a recent approach [39], we investigated the dimensional relations between factors within a population-based cohort of children with ASD instead of conducting a group comparison with a factorial design. The study used a structural equation modeling (SEM) framework, enabling the constructions of the latent factors EF and ToM derived from multiple measures, and therefore controlling the specific influences of each task or cognitive subdomain. Symptoms ASD and ADHD were indexed using multiple measures collected from multiple informants including parents, teachers, and the children themselves. Using this approach, we aimed to elucidate the specific and potentially clinically significant patterns of associations among those factors within the ASD population. We hypothesized specific associations between EF impairments and increased ADHD symptoms, and between ToM impairments and increased ASD symptoms.

## Methods

### Participants

The Special Needs and Autism Project (SNAP) is a population-based cohort of people with ASD who were first ascertained and characterized at the age of 10–12 years (wave 1; [50]). The children received research diagnoses of ASD according to the International Classification of Diseases (ICD-10; [51]), based on the Autism Diagnostic Interview-Revised (ADI-R; [52]) parental interview, the Autism Diagnostic Observation Schedule-Generic (ADOS-G; [53]) and IQ, language, and adaptive behavior. Of the original ASD sample, *N* = 131 (77%) had full-scale intelligent quotient (FSIQ) ≥ 50 and were followed up at the age of 14–16 years (wave 2). Of these 131 individuals, 19 declined to take part, 11 could not be contacted and one stated interest but could not take part before the end of the study. Thus, in total, 100 adolescents (*n* = 9 females) participated in the current study. The children did not differ from those who did not take part, although eligible (*n* = 31), on baseline measures of IQ, the ADOS-G and ADI-R total scores, the Social Responsiveness Scale (SRS; [54]) total score, the Diagnostic and Statistical Manual of Mental Disorders (DSM-IV) ADHD symptom numbers, and parent- and teacher-rated hyperactivity/inattention scores on the Strengths and Difficulties Questionnaire (SDQ; [55]) (*t*s = .32–1.5, *p*s = .14–.75). The follow-up was conducted in two sessions completed on average in 29 days (SD = 36 days; range = 1–259 days) with 94 participants completing the follow-up session within 2 × SD days from the mean. Tests were divided equally between 2 days, with IQ tests reserved for the first day of testing, in case of participant drop-out. For each testing day, a task order was fixed that enabled presentation of activities to be balanced (e.g., alternating between computerized and pen and paper tasks) and accommodate any constraints (e.g., tasks with a fixed duration). Half of the participants received the tasks in reverse order with some adjustment to account for task constraints. The study was approved by the South-East London Research Ethics Committee (05/MRE01/67). Informed consent was given by the parents and by the participant if their level of understanding was sufficient.

### Measures

Measures used in this study are described below and in the supplement. All measures were collected from the young people or parent/teacher over two waves of studies, when the young people were 10–16 years. The measures and timing (wave 1 or 2) are listed in Additional file 1: Table S1.

#### Cognitive measure

The Wechsler Abbreviated Scale of Intelligence (WASI; [56]) was chosen as a brief but reliable measure of general intellectual ability. The WASI consisted of four subtests, all contributed to an estimate of full-scale IQ (FSIQ).

#### ASD measures

ASD symptoms were indexed by (1) total algorithm score on the ADOS-G, (2) total algorithm score on the ADI-R, and (3) parent ratings on the SRS.

#### ADHD measures

ADHD symptoms were measured using (1) parent and teacher ratings on the hyperactivity domain of the SDQ, (2) parent report of inattention and hyperactivity/impulsivity symptoms frequency and their impact on everyday functioning on the Profile of Neuropsychiatric Symptoms (PONS; [57]), and (3) the number DSM-IV ADHD symptoms endorsed by parents on the Child and Adolescent Psychiatric Assessment interview (CAPA; [58]).

#### EF measures

The EF measures included in this study were (1) Opposite Worlds, part of the Test of Everyday Attention for Children [59] and (2) Luria Hand Game [60] as measures of inhibition; (3) Trail Making Test [61] and (4) a card sorting task [62] as measures of cognitive flexibility or switching; (5) a planning drawing task [63] as an index of planning; and (6) numbers (backward), taken from Children’s Memory Scale [64], as a measure of working memory. The measures were selected based on the ADHD literature and a previous model of EF [25, 65].

#### ToM measures

The ToM measures included in this study were (1) a combined False-Belief story (“The Chocolate Story”), a test of first- and second-order false-belief understanding [66, 67]; (2) Reading the Mind in the Eyes Task [68], an assessment of the ability of an individual to infer emotional state from photographic images of pairs of eyes; (3) Penny Hiding Game [69], a naturalistic nonverbal deception task; (4) Strange Stories Test [70], a verbal test consisting of short stories illustrating complex interaction involving lies, double bluffs, or persuasion; and (5) Frith-Happé Animated Triangles Task [71] that assesses spontaneous attribution of mental states towards animated geometric objects.

### Analytical plan

Data preparation and descriptive analyses were undertaken in STATA 11 [72]. Raw data were reversed when necessary, so higher scores reflected greater symptoms or difficulties. Box-Cox transformations were used to normalize skewed data (Table 2), and structural equation modeling (SEM) was used to model the relations among factors. Although the data were collected over 6 years, we modeled their associations within a single cross-sectional design. The analysis was divided in four steps. In step 1, the structure of the latent factors for EF and ToM was investigated using an exploratory factor analysis (EFA) with all indicators entered, employing a Geomin rotation. An EFA was thought more suitable than a confirmatory factor analysis for two reasons: (1) the factor structures of the cognitive domains indexed by the candidate EF and ToM measures were not completely clear as the two domains are usually explored separately (e.g., [25, 73]) and (2) individual neurocognitive measures are not “process pure” [34], often involving a mixture of cognitive domains. For this reason, we contrasted one (i.e., common cognitive factor) against two-factor (i.e., EF and ToM) predictor models, of which model fits were evaluated using χ^2^ statistics. A two-factor structure fits the EF and ToM factors better and was chosen. To improve the “purity” of the factors, indicators that cross-loaded, for example, significantly loaded to both the EF and ToM factors, or to the factor not expected a priori, and those or with factor loadings ≤ 0.4 were excluded in the first instance.

**Fig. 1:**
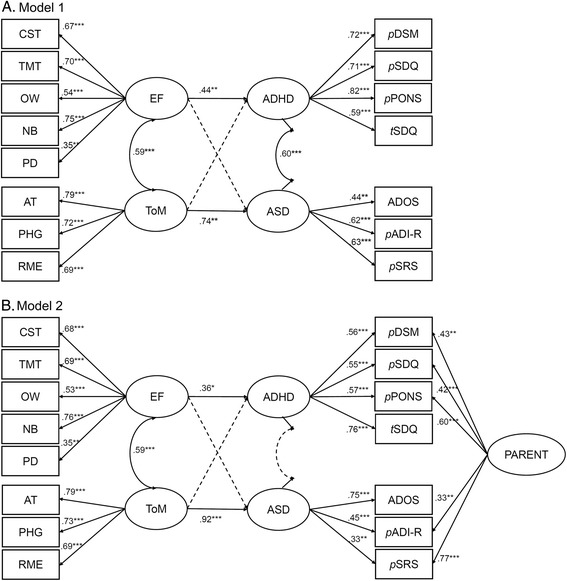
SEM models are presented here with nonsignificant paths shown on dotted lines. Nonsignificant paths in model 1 are between EF and ASD (*β* = − .11) and between ToM and ADHD factor (*β* = .07). In model 2, the nonsignificant paths are between EF and ASD (*β* = − .29), between ToM and ADHD (*β* = .16), and between ASD and ADHD (*r* = .03). List of abbreviations: *CST* card sort task, *TMT* Trail Making Test, *OW* Opposite Worlds, *NB* number backward, *PD* planning/drawing task, *AT* animated triangle, *PHG* penny hiding games, *RME* Reading the Mind in the Eye tasks, *EF* executive function, *ToM* theory of mind. Abbreviations for ASD or ADHD measures: *ADOS* Autism Diagnostic Observation Schedule, *ADI-R* Autism Diagnostic Interview-Revised, *SRS* Social Responsiveness Scale, *DSM* Diagnostic and Statistical Manual of Mental Disorders, *SDQ* Strengths and Difficulties Questionnaire, *PONS* Profile of Neuropsychiatric Symptoms. The prefix *p* on these behavioral measures indicates parent-based reports whereas the prefix *t* on the SDQ indicates a teacher-based report. The significant levels are *p < .05, **p < .01, and ***p < .001

In step 2, we built the SEM model 1 (Fig. 1a) to assess the relations between the neurocognitive (ToM and EF) and behavioral (ASD and ADHD) latent factors. The model was derived from the available data collected from the young people and parent/teacher over a period of 6 years when the young people were 10–16 years, taking the assumption of ADHD and ASD symptom persistence over the time window [74–76]. The data were modeled with EF and ToM “predicting” the symptom factors. However, the aim was not to test if cognitive factors causally underpin symptom domains, but rather to understand better the pattern of associations between cognition and behavior. The EF and ToM latent factors were allowed to correlate [43, 44] as were the ASD and ADHD factors [38, 39, 47, 77]. The EFA and SEM modeling was conducted in Mplus [78].

Inspection of the residual covariances suggested that accounting for shared information from parents could improve model fit. Therefore, in step 3, we introduced a parental reporting latent factor to account for shared informant influences from parents (model 2; Fig. 1b). The parental latent factor was indexed by symptom measures reported by parents (i.e., excluding the ADOS and the SDQ teacher report). The final form of model 2 was derived in step 4, by systematically removing nonsignificant pathways to increase parsimony and comparing the nested models iteratively. Since many individuals with ASD and ADHD have low IQ (e.g., [79, 80]), we examined in a sensitivity analysis whether the associations among factors remained after controlling for FSIQ in model 2. The ToM, EF, ASD, and ADHD latent factors were regressed on FSIQ in step 5 (Additional file 1: Figure S1A), and the associations between the neurocognitive and symptom factors were inspected. Mindful of the possible changes of ASD or ADHD traits from childhood to adolescence, in step 6, we conducted a sensitivity analysis by modeling the data collected from the children at age 14–16 years, excluding the teacher SDQ hyperactivity domain and DSM-IV ADHD symptoms from the ADHD factor, the total ADOS-G and ADI-R scores from the ASD factor, and the parental latent factor since all behavior scores were parent-rated. In this model, ASD symptoms were indexed by the observed SRS score. The analyses were repeated excluding data from children whose experimental sessions were separated by time window larger than 2 × SD from the mean to see if the extreme testing day separation could have impacted the findings. All models were fitted by maximum likelihood with robust standard error (MLR) which provides unbiased estimates for missing data with missingness assumed at random. The model fit for nested models was evaluated using the likelihood ratio chi-square, comparative fit index (CFI), and the Tucker-Lewis Index (TLI [81]; acceptable fit for both indices ≥ .90), the root mean square error of approximation (RMSEA [82]; acceptable fit ≤ .08). The Bayesian information criterion (BIC) and the Akaike information criterion (AIC) were provided in some cases to allow comparisons of nonnested models.

## Results

Descriptive statistics of measures are given in Table 1, and their bivariate correlations, with associated *p* values uncorrected for multiple comparisons, are in Table 2.

**Table 1:** Descriptive statistics of measures

		Mean (SD)	Range
IQ measures			
	FSIQ	84.3 (18.0)	50–119
	VIQ	80.8 (18.0)	55–120
	PIQ	90.4 (18.6)	53–126
EF indicators			
	Card sort task (98/100)^a^	7.2 (6.6)	1–36
	Luria Hand Game (97/100)^b^	2.8 (3.3)	0–15
	Trail making (88/100)^a^	63.4 (44.0)	13.4–257.1
	Planning/drawing (98/100)^a, b^	2.4 (1.7)	0–6
	Opposite Worlds (98/100)^a, b^	8.4 (7.5)	−3.7 – 47.4
	Numbers (99/100)^a, b^	7.3 (2.5)	0–12
ToM indicators			
	RME (94/100)^b^	14.0 (4.4)	6–25
	Penny hiding game^b^	2.3 (2.7)	0–14
	Strange stories (88/100)^b^	4.6 (2.1)	0–8
	Animated triangle (87/100)^a, b^	2.9 (0.9)	0–4.75
	False belief (99/100)^b^	3.3 (2.4)	0–8
ADHD symptom indicators			
	Parent SDQ hyperactivity (93/100)^a^	5.8 (2.5)	0–10
	Teacher SDQ hyperactivity (85/100)	5.5 (2.4)	1–10
	Parent PONS ADHD (89/100)^a^	10.7 (6.6)	0–27
	DSM-IV ADHD symptoms (73/100)	6.0 (3.6)	0–14
ASD symptom indicators			
	Parent SRS total raw score (92/100)	92.5 (29.3)	21–153
	ADI-R total (99/100)	21.5 (7.7)	5–41
	ADOS total	11.6 (6.1)	1–27

Notes. Descriptive statistics reported here are based on raw data

Abbreviations: *FSIQ* Full Scale IQ, *VIQ* Verbal IQ, *PIQ* Performance IQ, *RME* Reading the Mind in the Eye Tasks, *SDQ* Strengths and Difficulties Questionnaire, *PONS* Profile of Neuropsychiatric Symptoms, *SRS* Social Responsiveness Scale, *ADI-R* Autism Diagnostic Interview-Revised, *ADOS* Autism Diagnostic Observation Schedule

^a^Box-Cox transformed during analyses

^b^Reversed scores on this table

**Table 2:** Bivariate correlations among neurocognitive and symptom measures

	EF measures						ToM measures					ADHD measures				ASD measures	
	1	2	3	4	5	6	7	8	9	10	11	12	13	14	15	16	17
Card sort task (1)	**–**																
Luria Hand Game (2)	.28** (.05)	–															
Trail making (3)	.36*** (.12)	.26* (.11)	–														
Opposite Worlds (4)	.31** (.08)	.17 (.03)	.38*** (.28**)	–													
Numbers (5)	.51*** (.21*)	.35*** (.18†)	.47*** (.21**)	.37*** (− .20†)	–												
Planning/drawing (6)	.36*** (.21*)	.20† (.08)	.17 (.05)	.18† (.06)	.17† (− .02)	–											
Animated triangle (7)	.33** (.12)	.10 (.01)	.02 (− .13)	.14 (− .003)	.28** (.06)	.002 (− .13)	–										
Penny hiding game (8)	.27** (.06)	.45*** (.36***)	.17 (.04)	.26* (.15)	.30** (.12)	.14 (.02)	.37*** (.32**)	–									
RME (9)	.22* (− .19†)	.39*** (.25*)	.25* (.04)	.21* (.01)	.39*** (.11)	.06 (− .10)	.41*** (.25*)	.47*** (.37***)	–								
Strange stories (10)	.29** (− .07)	.22* (.07)	.18 (− .07)	.23* (.04)	.37*** (.07)	.25* (.14)	.20† (− .04)	.29** (.12)	.29** (− .003)	–							
False belief (11)	.63*** (.30**)	.53*** (.30**)	.32** (− .01)	.25* (− .05)	.46*** (.05)	.27** (.06)	.31** (.02)	.54*** (.44***)	.45*** (.11)	.43*** (.08)	–						
*p*PONS ADHD (12)	.16 (.06)	.10 (.05)	.21† (.16)	.23* (.18)	.26* (.19†)	.21† (.17)	.10 (.04)	.24* (.20†)	.11 (.02)	.01 (− .09)	.03 (− .13)	–					
*p*DSM ADHD (13)	.27* (.19)	.16 (.10)	.20 (.14)	.19 (.15)	.25* (.19)	.30** (.26*)	.08 (.03)	.10 (.04)	.09 (− .01)	.09 (− .005)	.13 (− .06)	.55*** (.54***)	–				
*p*SDQ ADHD (14)	.17 (.05)	.22* (.17)	.31** (.24*)	.21* (.15)	.28** (.20†)	.14 (.08)	.12 (.01)	.19† (.12)	.25* (.16)	.08 (− .04)	.08 (− .10)	.56*** (.54***)	.46*** (.44***)	–			
*t*SDQ ADHD (15)	.24* (.20†)	.28* (.24*)	.13 (.06)	.11 (.04)	.31** (.33**)	.14 (.09)	.12 (.05)	.30** (.27*)	.14 (.05)	− .14 (− .26*)	.22* (.16)	.41*** (.39***)	.50*** (.48***)	.38*** (.36**)	–		
*p*ADI-R (16)	.18† (.10)	.19† (.14)	− .03 (− .16)	− .05 (− .14)	.05 (−.05)	.14 (.10)	.29** (.25*)	.31** (.26*)	.03 (− .07)	.08 (.03)	.28** (.24*)	.16 (.14)	.35** (.33**)	.22* (.17)	.25* (.23*)	–	
ADOS (17)	.17† (.05)	.27** (.20*)	.05 (− .05)	− .02 (− .13)	.11 (−.02)	.19† (.12)	.38*** (.29**)	.42*** (.37***)	.33** (.29**)	.29** (.26*)	.36** (.24*)	− .01 (− .06)	− .17 (− .21†)	− .02 (− .09)	.14 (.12)	.34*** (.33**)	–
*p*SRS (18)	.17 (− .04)	.28** (.20†)	.27* (.11)	.14 (.03)	.18 (.007)	.12 (.01)	.15 (.03)	.25* (.16)	.29** (.15)	.20† (.06)	.20† (.05)	.50*** (.48***)	.36** (.32**)	.38*** (.33**)	.09 (.05)	.43*** (.40***)	.15 (.07)

Notes. Bivariate correlation coefficients are presented, with uncorrected *p* values for multiple comparisons. The coefficients in brackets are partialled for FSIQ. The prefix *p* on the measures indicates parent reports whereas the prefix *t* indicates a teacher report

Abbreviations of neurocognitive measures: *EF* executive function, *ToM* Theory of Mind, *RME* Reading the Mind in the Eye Tasks. Abbreviations for ASD or ADHD measures: *ADOS* Autism Diagnostic Observation Schedule Total Score, *ADI-R* Autism Diagnostic Interview-Revised, *SRS* Social Responsiveness Scale, *DSM ADHD* Diagnostic and Statistical Manual of Mental Disorders ADHD symptom numbers*, SDQ ADHD* Strengths and Difficulties Questionnaire ADHD domain, *PONS ADHD* Profile of Neuropsychiatric Symptoms ADHD domain

Significant levels †*p* < .1, *p < .05, **p < .01, and ***p < .001

### Step 1: EFA of predictor factors

The EFA of the ToM and EF indicators better fitted a two-factor (χ^2^[34] = 49.8, *p* = .04; CFI = .94; TLI = .91; RMSEA = .07) than a one-factor model (χ^2^[44] = 81.5, *p* = .0005; CFI = .87; TLI = .83; RMSEA = .09; Δχ^2^[10] = 32.5, *p* < .001). ToM and EF were correlated (*r* = .62, *p* < .05). Inspection of this model revealed that several tasks cross-loaded on the “other” neurocognitive domain (Table 3). The Luria Hand Game cross-loaded on ToM (factor loading = .49) and performances on the Strange Stories and False-Belief tasks cross-loaded on EF (factor loadings = .38 and .44, respectively). As they loaded on factors not expected a priori, they were thought to be less “pure” than other indicators and were removed from the subsequent SEM model. The model of the predictors with no cross-loading indicators still fit well to a two-factor model (χ^2^[13] = 16.2, *p* = .24; CFI = .98; TLI = .95; RMSEA = .04), but the correlation between EF and ToM was reduced from .62 to .40.

**Table 3:** Loading of the measures on factors EF and ToM

	With cross-loading indicators		Without cross-loading indicators	
	EF	ToM	EF	ToM
Card sort task	.75*	.003	.56*	.19
Trail making	.71*	− .07	.80*	− .09
Opposite Worlds	.47*	.01	.54*	.005
Luria Hand Game^a^	.17	.49*	–	–
Numbers	.66*	.06	.67*	.00
Planning/drawing	.47*	− .09	.37*	.06
Animated triangles	.004	.72*	− .01	.94*
Penny hiding game	− .12	.84*	.13	.61*
RME	.05	.65*	.26	.50*
Strange stories^a^	.38*	.26	–	–
False belief^a^	.44*	.46*	–	–

Abbreviation: *RME* Reading the Mind in the Eyes Task

^a^Measures which cross-loaded on factors not expected a priori. We excluded these measures from the final model to separate the predictors

*Significant at *p* = .05 level

### Step 2: The associations among EF, ToM, and ASD and ADHD symptoms

Model 1 (Fig. 1a) approached an acceptable fit (χ^2^[84] = 151.7, *p* < .0001; CFI = .82; TLI = .77; RMSEA = .09; AIC = 6063.9; BIC = 6196.8). Critically, paths between EF and ADHD (*β* = .44, *p* = .005), ToM and ASD (*β* = .74, *p* = .007), EF and ToM (*β* = .59, *p* < .001), and ASD and ADHD (*β* = .60, *p* = .021) were significant. Those between EF and ASD (*β* = − .11, *p* = .7) and ToM and ADHD (*β* = .07, *p* = .6) were not.

### Step 3: Adding a parental latent factor

Accounting for the shared parental information resulted in model 2 that approached the threshold of acceptability (χ^2^[79] = 114.3, *p* = .006; CFI = .90; TLI = .87; RMSEA = .067; AIC = 6039.8; BIC = 6185.7). The paths of interest between EF and ADHD symptoms (*β* = .36, *p* = .049) and ToM and ASD symptoms remained (*β* = .94, *p* < .001), and the paths between EF and ASD and ToM and ADHD were nonsignificant. The correlation between ASD and ADHD became nonsignificant (*r* = .03, *p =* .94), indicating that the correlation between these symptom domains was partially accounted for by the shared variance of the parent ratings.

### Step 4: Derivation of the final model

**Fig. 2:**
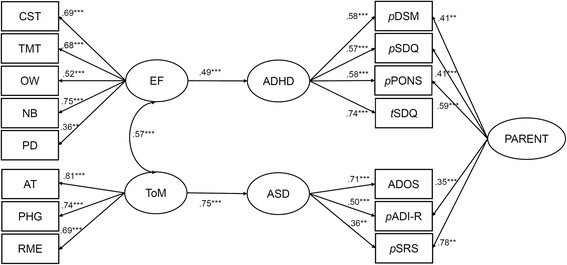
SEM final model is derived from model 2 with nonsignificant paths removed. List of abbreviations: *CST* card sort task, *TMT* Trail Making Test, *OW* Opposite Worlds, *NB* number backward, *PD* planning/drawing task, AT animated triangle, *PHG* penny hiding games, *RME* Reading the Mind in the Eye tasks, *EF* executive function, *ToM* theory of mind. Abbreviations for ASD or ADHD measures: *ADOS* Autism Diagnostic Observation Schedule, *ADI-R* Autism Diagnostic Interview-Revised, *SRS* Social Responsiveness Scale, *DSM* Diagnostic and Statistical Manual of Mental Disorders, *SDQ* Strengths and Difficulties Questionnaire, *PONS* Profile of Neuropsychiatric Symptoms. The prefix *p* on these behavioral measures indicates parent-based reports whereas the prefix *t* on the SDQ indicates a teacher-based report. The significant levels are *p < .05, **p < .01, and ***p < .001

The final model (Fig. 2) was derived from model 2 with nonsignificant paths systematically removed. The nonsignificant correlation between ASD and ADHD was the first to be removed, as it ceased to be significant upon the addition of parental factor. This resulted in a model of which fit was not significantly worse than the full model (Sattora-Bentler scaled Δχ^2^[1] = .007, *p* = .93; CFI = .91; TLI = .88; RMSEA = .064). The removal of a second nonsignificant path between EF and ASD factors also did not significantly change the last model fit (Sattora-Bentler scaled Δχ^2^[1] = 1.35, *p* = .25; CFI = .91; TLI = .88; RMSEA = .064). The final removal of the nonsignificant path between ToM and ADHD also did not worsen the model fit (Sattora-Bentler scaled Δχ^2^[1] = .80, *p* = .37; CFI = .91; TLI = .88; RMSEA = .064; AIC = 6037.0; BIC = 6175.0). This model showed that EF impairments were associated with increased ADHD symptoms (*β* = .49, *p* = .001), ToM impairments were associated with ASD symptoms (*β* = .75, *p* < .001), and EF and ToM were correlated (*r* = .57, *p* < .001).

### Step 5: Sensitivity analysis of a model including FSIQ

By regressing EF, ToM, ASD, and ADHD in full model 2 on FSIQ and removing nonsignificant paths, we arrived at the final parsimonious model (χ^2^[95] = 138.7, *p* = .002; CFI = .91; TLI = .88; RMSEA = .07; AIC = 6811.5; BIC = 6960; Additional file 1: Figure S1B), which retained the specific relations between EF and ADHD (*β* = .40, *p* = .002) and ToM and ASD (*β* = .75, *p* < .001), while controlling for the association between higher IQ and less impairment in EF (*β* = − .84, *p* < .001) and ToM (*β* = − .65, *p* < .001).

### Step 6: Sensitivity analysis using 14–16-year-old data

The fit for the parsimonious model including only the adolescent data was excellent (χ^2^[51] = 52.7, *p* = .41; CFI = .99; TLI = .99; RMSEA = .018; AIC = 3914.5; BIC = 4010.9; Additional file 1: Figure S2B), consisting of specific relations between EF and ADHD (*β* = .38, *p* = .005) and between ToM and observed ASD symptoms (*β* = .39, *p* < .001), while controlling for the association between IQ to EF (*β* = − .84, *p* < .001) and ToM impairments (*β* = − .72, *p <* .001). The model structure was also preserved when children whose two testing days were separated by more than the 2 × SD days of the mean were excluded from the analyses (see Additional file 1).

## Discussion

Although ADHD symptoms are frequently seen in people with ASD, the basis for this association remains poorly understood. This study explored the specificity of relations among executive dysfunction, ToM impairments, and the ASD and ADHD traits using the framework of SEM, in a population-based sample of children with ASD. The primary findings of the study are that poorer EF ability is specifically associated with increased ADHD symptoms, accounting for its association with variation in ToM performance and ASD symptoms. Furthermore, impairments in ToM are specifically associated with ASD symptoms. Taken together, the findings show that ADHD and ASD symptoms in adolescents with ASD have dissociable neurocognitive correlates. The secondary findings are that the observed correlation between ASD and ADHD symptoms in the sample can be explained by shared source of reporting from parents and the correlation between EF and ToM. Finally, the specific relations between each cognitive domain and the behavioral symptoms are retained even when controlling for IQ.

### Specific associations between cognitive domains and symptoms

Our model shows that the EF impairments among individuals with ASD are associated with increased ADHD symptoms. This is in line with findings from previous group comparisons reporting increased EF difficulties among children with dual diagnoses of ASD and ADHD compared to children with ASD alone [11, 36–38] and the associations reported between EF deficits and increased ADHD symptoms in ASD samples [39, 83]. In addition, the association between EF impairments and increased ADHD symptoms in this ASD sample is consistent with findings in other samples of individuals with ADHD (e.g., [8, 26, 27, 84]) and in the general populations (e.g., [85–87]). Importantly, we did not detect a significant association between ToM impairments and ADHD symptoms in the model, consistent with a recent meta-analytic finding showing that ToM difficulties are present to a much lesser extent in ADHD than ASD populations [9]. The specific relation between EF and ADHD symptoms in this context may provide an explanation for the mixed findings of EF difficulties in ASD (e.g., [17, 29, 30]), in that, EF difficulties are perhaps more likely to be found among people with ASD who have co-occurring ADHD.

The SEM model also shows that mentalizing abilities are specifically associated with the severity of ASD and not ADHD symptoms. This is consistent with results from previous studies [88–90] and supports the view that ToM impairments are specifically linked to ASD symptoms. These findings contrast with some studies that fail to find an association between ToM and everyday social behavior in people with ASD (e.g., [91–93]), perhaps due to these studies’ reliance on specific measures such as the false belief test, which may not fully capture the breadth of socio-cognitive and perceptual processes related to ToM [92, 94, 95]. We have addressed this potential limitation by using a multi-measure approach to better capture the ToM construct.

Contrary to previous findings of associations between performance of a variety of EF tasks and ASD symptoms [39, 44, 92, 96] and specifically between cognitive flexibility and repetitive behavior symptoms (e.g., [31–34]), no association was found between EF impairments and ASD traits in the SEM model. There could be several explanations for this finding. Firstly, none of these previous studies considered the association between EF and ASD after controlling for ToM impairments. Therefore, the association between EF and ASD symptoms might have been observed in those studies because the covariation of ToM and ASD or EF and ADHD symptoms was not accounted for. Secondly, many studies reporting the associations between EF impairments and ASD symptoms analyzed these relations separately between tasks that may tap different aspects of EF. Thus, the correlations found between task performance and ASD symptoms, notably between cognitive flexibility performance and repetitive behavior symptoms, may reflect relations between properties that are specific to the task or the EF subdomain, rather than the underlying common EF factor, with the ASD symptoms. Lastly, as learnt from the finding in relation to the Luria Hand Game, performance on a task that is traditionally an inhibitory measure may load onto ToM instead of EF factors, thus more associated with ASD symptoms. This could be because the task triggers mentalizing processes when the subjects attempt to guess which hand gesture the experimenter would be giving next. Such interpretation is in line with the Triple-I hypothesis [97], which argues that EF impairments in ASD might be a by-product of mentalizing deficits in the population.

### A shared parental rating factor partially accounts for the correlation between ASD and ADHD traits

Several previous studies have found that individuals with ASD (and co-occurring ADHD symptoms) have more severe ASD traits relative to those with ASD alone, judged from parent-rated questionnaires such as the SRS [38, 47, 49]. Autistic trait measures have less specificity when applied to children with ASD and additional behavioral or emotional problems, including ADHD [98, 99]; thus, it is possible that past reports of an association between ASD and ADHD traits were due to a systematic instrument bias, in which those with additional ADHD traits also receive higher ratings of autistic traits. The association between ADHD and ASD trait severity was noticeably absent in previous studies; however, when ASD traits were measured using measures such as the ADI-R, ADOS [38, 47, 48, 75] or clinical symptom counts [74]. Unlike the SRS, which is parent questionnaire, measures such as the ADI-R and clinical symptoms involve clinical judgment, and in the case of ADOS, direct observation of the children. Therefore, the association between the ASD and ADHD traits found in previous studies might be partially dependent on the source and type of reporting.

Indeed, the final SEM model showed that information obtained from the same source (i.e., parents in this case) on ASD and ADHD traits moderated their correlation. That is, ratings by the same informant on different measures are more highly correlated than those from different informants. There are multiple possible explanations for this finding. Informants, in this case, parents, may have a specific response style, such as a tendency to rate all behaviors as high or low, that influences their responses across measures [100]. Furthermore, children’s behavior may differ across settings [101, 102]. As in many studies, parents were the predominant source of information regarding symptoms in this study. Because no other information source was shared across ASD and ADHD measures, it was not possible to test whether these effects are specific to parents or applies to other sources, such as teachers or direct observation. Nevertheless, our findings underline the importance of obtaining multiple sources of information [103].

### Explaining the co-occurrence of ADHD symptoms among ASD children

In line with the comorbidity literature, our model showed that artifactual and non-artifactual mechanisms could explain the increased co-occurrence of ADHD traits in the ASD children population. Firstly, ADHD traits might be reported at increased rates, presumably in those with more severe ASD, due to shared reporting source effects from parents. These cases, which do not express true comorbidity, one hopes are few and can be differentiated from true comorbidity cases during clinical observations, corroborated by non-parental source of information. Aside from this artifactual reason, our model provides preliminary supports for the cognitive mechanisms underlying the true comorbidity of ADHD traits in children with ASD. We have shown in this study that the cognitive correlates of the ASD and ADHD traits in children are dissociable, which supports the additive model for the co-occurrence of ADHD traits on ASD symptoms. Furthermore, from our model, the co-occurrence of the separable ASD and ADHD traits could be explained partially by the moderate correlation between EF and ToM impairments (.56), indicating that individuals with both ASD and ADHD symptoms constitute those who are “doubly hit” by both EF and ToM impairments.

Due to the limitations posed by the available data, the support our model gives to the additive model is preliminary. To satisfactorily adhere to the additive comorbidity model, we believe that the ADHD traits found in our ASD population must be phenomenologically equivalent, both in presentations and their associations to the cognitive factors, with symptoms found in the pure ADHD population. While previous findings suggest that ADHD symptoms in clinical ASD and ADHD populations have similar presentations (inattentive, hyperactive, and combined [104, 105]) with only subtle differences in few symptoms [106, 107], and although the association found between executive dysfunction and ADHD traits in our sample is consistent with other findings in ADHD population (e.g., [8, 26, 27, 84]), neither could be directly observed in this study due to the lack of ADHD comparison sample.

### Strengths and limitations

The inclusion of a well-characterized sample of children with ASD is an advantage for this study. Although the sample is not ASD population representative due to the exclusion of individuals with IQ below 50, it is still population-based and is free from clinical sampling biases. In fact, the comparisons we made between eligible individuals who did and did not take part in the study suggested that the study participants had representative ASD and ADHD traits for ASD children with IQ of 50 and above, therefore substantively extending previous neurocognitive findings in this topic which typically excluded children with learning disability. The inclusion of multiple measures from multiple informants gives an advantage over the typical approach of using single measures. The study includes multiple measures of ADHD psychopathology including the SDQ, a well-recognized screening instrument for children and adolescents, and the diagnostic instrument CAPA. Furthermore, the ADOS and the ADI-R, used to index autistic traits in the model, are considered “gold-standard” research instruments for assessing ASD [108].

One limitation of the study is that factors were modeled with EF and ToM predicting the ADHD and ASD symptoms, in line with the idea that EF and ToM deficits are endophenotypes that mediate the link between genes and behavioral symptoms (see [10] for alternative models that do not assume this directionality). However, the study design does not allow a strong test of causal direction. Therefore, the links between factors in the present model are best understood as associations rather than predictions. Secondly, we have modeled associations between factors indexed by measures collected over a 6-year period, thus assuming the stability of behavioral traits within the time window, which was supported by findings of persisting ASD and ADHD traits from childhood to adolescents reported in previous studies [74–76]. Furthermore, the additional analyses including only data collected during the adolescent years preserve the specificity of relations between the factors EF and ADHD and between ToM and observed ASD symptoms in the model. Finally, the small number of girls in the study may limit the generalizability of the results among females with ASD.

## Conclusions

This study adds to the growing literature that explores the cognitive underpinnings of ADHD symptoms in the ASD population. We found that there is a specific association between EF and ADHD symptoms that remained even after controlling for their associations with variations in ToM abilities and ASD symptoms, which supports the additive hypothesis of ADHD symptoms of ASD in the comorbid cases. Within the clinical context, this finding improves our understanding of how impairments in distinct cognitive domains contribute to the phenotypic variations of ASD, which often include additional presentation of ADHD. Our findings also suggest similarities in the cognitive correlates of ADHD symptoms in ASD as in pure ADHD [35, 39], although this remains to be tested by comparing the model in both ASD and ADHD populations. Importantly, the association between EF and ToM impairments could provide a partial explanation for the co-occurrence of ADHD symptoms in ASD. Finally, shared reporting effects from parents should be considered when examining ADHD symptoms in the ASD population.

## Additional file

Additional file 1: Table S1. Measures, completers, and wave of investigations. **Figure S1.** Controlling for IQ in the final model. Latent factors EF, ToM, ASD, and ADHD were regressed on IQ in the full final model (Figure A), nonsignificant paths were represented by dotted lines. **Figure S2.** Model including only measures from collected from the adolescents at the age of 14–16 years old. (DOCX 488 kb)

## Abbreviations

ADHD: Attention-deficit/hyperactivity disorder
ADI-R: Autism Diagnostic Interview-Revised
ADOS-G: Autism Diagnostic Observation Schedule-Generic
AIC: Akaike information criterion
ASD: Autism spectrum disorder
AT: Animated triangle task
BIC: Bayesian information criterion
CAPA: Child and Adolescent Psychiatric Assessment
CFI: Comparative fit index
CST: Card sorting task
DSM: Diagnostic and Statistical Manual of Mental Disorders
EF: Executive function
EFA: Exploratory factor analysis
FSIQ: Full Scale IQ
ICD-10: International Classification of Diseases
NB: Numbers (backward)
OW: Opposite Worlds
PD: Planning drawing task
PHG: Penny hiding game
PIQ: Performance IQ
PONS: Profile of Neuropsychiatric Symptoms
RME: Reading the Mind in the Eyes
RMSEA: Root mean square error of approximation
SDQ: Strengths and Difficulties Questionnaire
SEM: Structural equation modeling
SNAP: Special Needs and Autism Project
SRS: Social Responsiveness Scale
TLI: Tucker-Lewis Index
TMT: Trail Making Test
ToM: Theory of mind
VIQ: Verbal IQ
WASI: Wechsler’s Abbreviated Scale of Intelligence
WISC: Wechsler Intelligence Scale for Children
