# Supplementary material for: Specificity of executive function and theory of mind performance in relation to attention-deficit/hyperactivity symptoms in autism spectrum disorders
Source: Mol Autism. 2017 Nov 9;8:60. doi: 10.1186/s13229-017-0177-1 (PMC5680830; doi:10.1186/s13229-017-0177-1)
Supplement: Additional file 1: Table S1. — Measures, completers, and wave of investigations. Figure S1. Controlling for IQ in the final model. Latent factors EF, ToM, ASD, and ADHD were regressed on IQ in the full final model (Figure A), nonsignificant paths were represented by dotted lines. Figure S2. Model including only measures from collected from the adolescents at the age of 14–16 years old. (DOCX 488 kb) [file 13229_2017_177_MOESM1_ESM.docx]

# Additional file

## Measures

All measures used in the model were collected from the young people or parent/teacher informants over two waves of studies, when the young people were between 10 and 16 years. The measures and timing (Wave 1 or 2) are presented in Table S1. Descriptions of some measures are included below.

| Table S1: Measures, Completers, and Wave of Investigations | | | |
| --- | --- | --- | --- |
| Domains | Measures | Completed by | Wave |
| IQ | WASI | child | 2 |
| ASD symptoms | ADI-R | parent | 1 |
|  | ADOS-G | child | 1 |
|  | SRS | parent | 2 |
| ADHD symptoms | SDQ | parent,  teacher | parent (2),  teacher (1) |
|  | PONS | parent | 2 |
|  | CAPA^a^ | parent | 1 |
| EF | Card sorting task | child | 2 |
|  | Luria hand game |  |  |
|  | Trail making |  |  |
|  | Planning/drawing |  |  |
|  | Opposite worlds |  |  |
|  | Numbers |  |  |
| ToM | Reading the mind in the eyes | child | 2 |
|  | Penny hiding game |  |  |
|  | Animated triangle |  |  |
|  | Strange stories |  |  |
|  | False belief |  |  |
| Wave 1 investigation took place when the children were 10-12 years old whereas Wave 2 investigation took place when they were 14-16 years. ^a^The CAPA interviews were conducted with parents when the children were 10-14 years. List of abbreviations: WASI = Wechsler’s abbreviated scale of intelligence. ADI-R = Autism Diagnostic Interview-Revised, ADOS-G = Autism Diagnostic Observation Schedule-Generic, SRS = Social Responsiveness Scale, and SDQ = Strengths and Difficulties Questionnaire, PONS = Profile of Neuropsychiatric Symptoms, CAPA = Child and Adolescent Psychiatric Assessment. | | | |

## ADHD Measure

**Profile of Neuropsychiatric Symptoms (PONS).** The PONS [1] is 60-item measure of psychiatric symptoms in children and adolescents, with special application to those with developmental disorders. Items evaluate both the frequency and the impact of individual symptoms. There are six items concerning ADHD symptoms, divided into symptom domains of attention, hyperactivity and impulsivity. Each domain asks about the symptom’s frequency (one item) and its impact on everyday functioning (one item) and each item is rated on a Likert scale (0-6). The sensitivity and specificity of the PONS was 92% and 91%, respectively, for screening children with neuro-developmental disability (ASD or ADHD) among 147 children aged 5-18 years with neuropsychiatric disorders, including (n = 111) with ADHD, (n = 96) with ASD, (n = 59) with oppositional defiant disorder or conduct disorder, (n = 31) with bipolar or psychosis, (n = 80) with anxiety or depressive disorder, (n = 38) with developmental coordination disorder, (n = 36) with obsessive compulsive disorder and/or tics (the children may meet several psychiatric disorders); and over 900 typically developing children from the general population. There are no ASD-specific psychometric evaluations of the PONS currently. An early version of the PONS was used in this study [2] where item scores ranged from 0 to 5, yielding a total score ranging from 0 to 30.

## EF Measures

**Card sorting task.** The card sorting task [3] is a child-friendly adaptation of the Wisconsin Card Sort Task [4]. In this task, participants were introduced to three characters and were given a deck of 64 cards illustrating single objects varying in colour (red/blue; yellow/green; black/pink), shape (squares/hearts; stars/moons; and smiley faces/lightning) and size (small/large). At the start of the game, the participants were told that each character favoured some cards over others with a specific rule the participants must solve. The experimenter picked one card at a time and asked the participants if it was the character’s favourite card. The experimenter gave feedback (i.e., “right” or “wrong”) for each answer provided by the participants, and then put the character’s favourite card face down on one pile and the disliked cards on another. After six consecutive correct sorts or after 20 sorts elapsed, another character was introduced to the participants and the sorting rule changed without explicit mention of a rule switch. The rules for the card sorting were counterbalanced and the participant’s decision on the first sort was always taken as the correct answer. The total number of sorting errors was used to indicate deficits in EF.

**Luria Hand Game.** The Luria hand game [5] consists of three stages: a pre-test, a practice run and a test. In this game, the experimenter displayed two types of hand shape with their right hand, a fist or, alternatively, a finger-pointing shape. Participants responded with an identical or the alternate hand shape (i.e., by showing a fist when the experimenter showed a finger-pointing shape) depending on the experimental block. In the pre-test, identical hand shape was required for six consecutive trials. In the practice run, the alternative hand shape was requested instead, and this trial stops after four consecutive correct responses. During the test, the experimenter showed a sequence of 15 trials of either hand shapes, and the participants were instructed to show the alternative hand shape in response. Between trials, the participants and experimenter hide their hands behind their back. The order of the sequence was fixed across participants. The number of correct responses on a first attempt indexed the inhibitory function ability. We used reversed scores in this study to operationalise inhibitory deficits.

**Trail Making Test.** Trail making [6] is a measure of switching or cognitive flexibility. It is a pen-and-paper task that requires a participant to draw a path connecting circles labelled with numbers 1-25 and letters A-Y in a specific order. The experimenter administered this task in three blocks. In the first block, the participant was asked to join circles in numerical order (i.e., 1-2-3 and so on), then in alphabetical order (i.e., A-B-C and so on) for the second, and in alternating order of numbers and letters (i.e., 1-A-2-B-3- and so on) for the third. The difference between the time taken to complete the last and the first trials indexed switching ability and higher scores indicated poorer performance.

**Planning/drawing task.** The planning/drawing task [7] consists of several blocks of copy-drawing and “modification” drawing trials. In the first trial, the participants were given an illustration to copy fully. In the following trial, the participants were required to do the same drawing with some additional features. For instance, in the first trial the participants were asked to copy a drawing of a snowman with an open mouth whereas in the second trial, they were asked to draw it again and with teeth in the snowman’s mouth. To complete this task successfully, the participants must plan prior to the task, e.g., increasing the size of key parts such as the snowman’s mouth, to accommodate some teeth. Evidence of prior planning earned the participants the “allowance scores”, judged from the comparison between drawings from the copy-drawing and the modification-drawing trials [7]. The score ranged from 2 points for clear and effective allowance, 1 point for clear but noneffective allowance, and 0 point for no allowance at all. Scores were reversed so that higher allowance scores indexed poorer planning corresponding to an EF deficit.

**Opposite Worlds.** This task is part of the Test of Everyday Attention for Children (TEA-Ch; [8]) and is a measure of inhibition. During this task, participants followed a trail consisting of the numbers “1” and “2” on a piece of paper and they were instructed to call out “one” or “two” as they passed each number. There were two task conditions, which were “same worlds” and “opposite worlds” to be completed twice each. In the same-world condition, the verbal call outs corresponded to the numbers, while in the opposite-world condition the participants were told to reverse the call outs and vocalise the opposite number. The difference of RT (seconds) to complete the opposite-world versus the same-world condition is a measure of inhibitory function, where a higher score indexed poorer performance.

**Numbers.** Numbers is a subtest of the Children’s Memory Scale [9]. In this task, the experimenter gave a sequence of one-digit numbers verbally to the participants at a rate of one number per second. The participants must then recall and repeat these sequences as accurately as possible. The experimenter started by giving two-digit sequences, then adding one digit in the next trial either until the participants failed to repeat the correct sequences twice within the span, or until the maximum nine-digit sequences were completed. The task was then repeated, but now the participants must repeat the digits in reversed order (digit spans ranging from 2-8). The number of correct trials was used as a measure of performance of working memory, and the scores were reversed to reflect difficulties.

## ToM Measures

**Reading the Mind in the Eyes.** The child version of the task [10] involves 28 photos of the eye regions of various individuals in different social contexts. Each photo was presented with four choices of adjectives, one of which was consistent with the feelings reflected in the pair of eyes. Each correct answer was given a score of one thus the participants could earn a maximum score of 28. A reversed score was used to operationalise the ToM deficits.

**Penny Hiding Game.** The penny hiding game [11] is a naturalistic and nonverbal deception task. This task started with a training phase where the experimenter hid a penny in one fist with both hands obscured behind their back. The experimenter then presented both fists to the participants who had to guess where the penny was hidden. During the training phase, the experimenter hid the penny six consecutive times from the participants. Then, the participant took his turn to hide the penny for the next six trials and deceive the experimenter. Error scores were given for failures to carry out the deception, which were systematically coded into five categories: (1) Failing to keep hands out of sight when hiding the penny, (2) telling the experimenter where the penny was, (3) using one hand for hiding or presenting, (4) holding the hands open when presenting, or (5) committing display error such as giving unintentional clues by having different grips of the clenched fist. More than one mistake could occur within each trial, giving a possible maximum score of five per trial and a total error score ranging from 0-30 for the entire task, where higher score reflected increased ToM difficulties.

**Strange Stories Test.** The strange stories test [12] consists of several short stories depicting several complex interactions involving lies, double bluffs or persuasion. The stories were read to the participants and were also presented in written form with illustrations. Four stories involving ToM and two “physical” stories were included in this test. The 0-1-2 scoring system described by Happé [12] was used where a score of zero was given to incorrect or “don’t know” responses and a score of two was given to children who gave fully and explicitly correct answers. The total score ranged from zero to eight and was a sum of the four ToM items that indexed ToM ability. Reversed scores were used as indicator of ToM deficits.

**Frith-Happé Animated Triangle.** This animated task [13] consists of six video clips depicting two triangles moving in a goal-directional manner (two clips) or in an interaction involving mental state attribution with one another (four clips). Participants were instructed to give verbal accounts of triangles action for each clip while being audio-recorded by the experimenter. The participants’ attributions of mental states towards the triangles were rated as the “intentionality scores”, ranging from zero to five, whereas the correct identification of the animation’s contents was rated as the “appropriateness scores”, ranging from zero to two, averaged across the number of clips [14]. Seventy-two of 129 verbal descriptions (56%) were coded by two raters independently, where high intra-class correlations (.82-.98) were found, indicating good reliability. The intentionality scores for each scenario were used as measure of ToM ability. Scores were reversed to index ToM deficits.

**False Belief (FB).** This measure consisted of one combined 1^st^ and 2^nd^ FB task (“The Chocolate Story”, adapted from stories used in previous studies [15, 16]), and another more complex 2^nd^ order FB task [17]. During the task, the experimenter presented a story with an accompanying cartoon to the participants. In the Chocolate Story, Mary and John hid some chocolate in a fridge together, but John then removed the chocolate in Mary’s absence. The experimenter asked the participants where they think Mary would look for the chocolate, followed by a “justification question” where the experimenter asked why Mary looked for the chocolate there, and a “control question” asking where John hid the chocolate. Subsequently, the experimenter added that Mary had in fact watched John removed the chocolate into the bag although John did not see her watching. In the same manner as the first part of the test, the participants were asked questions about where John thought Mary would look for the chocolate followed by a justification and control questions. In the 1^st^ order story, the participants were given the score one each for passing the FB and the justification question (score range 0-2). In the 2^nd^ order story, the participants would score one for passing the FB question and up to two for passing the justification question (i.e., the score one is given for correct justification, and the score two for both correct justification and use of mental state language). The participants would get a default zero score if failing on the FB question and they could earn up to five points in this first story. In the second 2^nd^ order FB task [17], the experimenter read out a story about Peter and Jane, who went to two shops to find Peter a new coat and planned to purchase it that evening. They later found separately that the coat Peter had wanted has sold out. The experimenter then asked the participants where Jane thought Peter has gone to buy his coat. This was followed by a justification question why they think Jane think so, producing a score ranging from 0-3. Further, the experimenter also asked a “memory question”, asking the participant in which shop Peter saw the coat he liked, although the score for this question did not contribute to the final performance score, which was only based on the false-belief questions and their justifications. Overall, the combined summary score ranged from 0-8 and was reversed to indicate poorer ToM ability.

## Sensitivity analysis 1: A model including FSIQ.

Regressing EF, ToM, ASD, and ADHD factors on FSIQ (Model 1; Figure S1) resulted in a model with near-acceptable fit (χ^2^[90] = 128.2, *p* = .005; CFI = .92; TLI = .89; RMSEA = .065; AIC = 5946.9; BIC = 6102.9). In this model, both EF (*β* = -.85, *p* < .001) and ToM (*β* = -.68, *p* < .001) regressed on FSIQ significantly. Paths of critical interests between EF and ADHD symptoms (*β* = .89, *p =* .005) and between ToM and ASD symptoms (*β* = .96, *p <* .001) retained their significance. They confirm the specificity of relations from the cognitive domains to behavioral symptoms after controlling for IQ. However, the model also suggested that the residual effect of FSIQ on ADHD symptoms were positive (*β* = .77, *p* = .004, i.e., higher IQ predicted higher ADHD symptoms when the associations between EF, ToM, ASD symptoms and IQ were controlled). A positive but nonsignificant residual effect of FSIQ was similarly found on ASD symptoms (*β* = .10, *p* = .73). These unlikely relations most possibly occurred because the associations between IQ and each set of behavioral symptoms were relatively weaker than the associations between IQ and each cognitive domain. For this reason, we turned into a data-driven method for deriving parsimonious model including IQ.

All directional paths were replaced by correlational pathways initially. Over a sequence of models, the largest and significant correlational pathways between the neurocognitive and behavioral factors were set to directional paths while nonsignificant correlational/partial correlations were removed until no further significant partial correlations remained. The effect of IQ was then examined on full Model 2. Correlations were found between ToM and ASD (*r* = 0.77, *p* < .001), FSIQ and ASD (*r* = -.37, *p* = .005), EF and ADHD (*r* = .45, *p* < .001), EF and ToM (*r* = .61, *p* < .001), ToM and ADHD (*r* = .36, *p* = .002), EF and FSIQ (*r* = -.85, *p* < .001), marginally between EF and ASD (*r* = .29, *p* = .054) and FSIQ and ADHD (*r* = -.22, *p* = .059), and not significantly between ASD and ADHD (*r* = .24, *p* = .23). The model was re-run specifying the path from ToM and ASD as predictive pathway, removing the nonsignificant pathways between ASD and ADHD and between FSIQ and ADHD and leaving the remaining paths as correlations. This resulted in a model of nearly acceptable fit (χ^2^[91] = 127.2, *p* = .01; CFI =.92; TLI =.90; RMSEA = .063), in which ToM predicted ASD (*β* =.76, *p* < .001). The correlation between EF and ADHD (*r* = .44, *p* = 0.001) remained significant but those between EF and ASD (*r* = -.27, *p* = .20) and IQ and ASD (*r* = .22, *p* = .16) became nonsignificant. IQ was significantly correlated with EF (*r* = -.85, *p* < .001) and ToM (*r* = -.67, *p* < .001). Furthermore, EF and ToM were correlated with each other (*r* = .60, *p* < .001). The next model was accomplished by setting the correlational path between EF and ADHD into predictive path, and by removing the correlational paths from ASD to EF and IQ. In this model (χ^2^[93] = 129.5, *p* = .01; CFI =.92; TLI =.90; RMSEA = .063), EF remained correlated with ToM (*r* = .57, *p* < .001) and IQ (*r* = -.85, *p* < .001), and ToM remained correlated with IQ (*r* = -.64, *p* < .001), although the correlational paths from ADHD to ToM (*r* = .11, *p* = .39) and IQ (*r* = .18, *p* = .063) became nonsignificant. The penultimate model was accomplished by setting the correlation between IQ to ToM and EF to predictive paths, and removing the correlations from ADHD to ToM and IQ, resulting in a model (χ^2^[94] = 137.8, *p* = .002; CFI =.91; TLI =.88; RMSEA = .07) where EF were associated with ADHD (*β* = .40, *p* = .002), ToM with ASD (*β* = .75, *p* < .001), and FSIQ with EF (*β* = -.84, *p* < .001) and ToM (*β* = -.65, *p* < .001), but EF and ToM were no longer correlated (*r* = .09, *p* = .59). The final parsimonious model (χ^2^[95] = 138.7, *p* = .002; CFI =.91; TLI =.89; RMSEA = .07; AIC = 6811.5; BIC = 6960.0; Model 2, Figure S1) was achieved by removing the correlation between EF and ToM. The final parsimonious model consisted of specific relations between EF and ADHD (*β* = .40, *p* = .002) and between ToM and ASD (*β* = .75, *p* < .001), while controlling for the significant effects of increased IQ on reduced impairments in EF (*β* = -.84, *p* < .001) and ToM (*β* = -.65, *p* < .001).

| 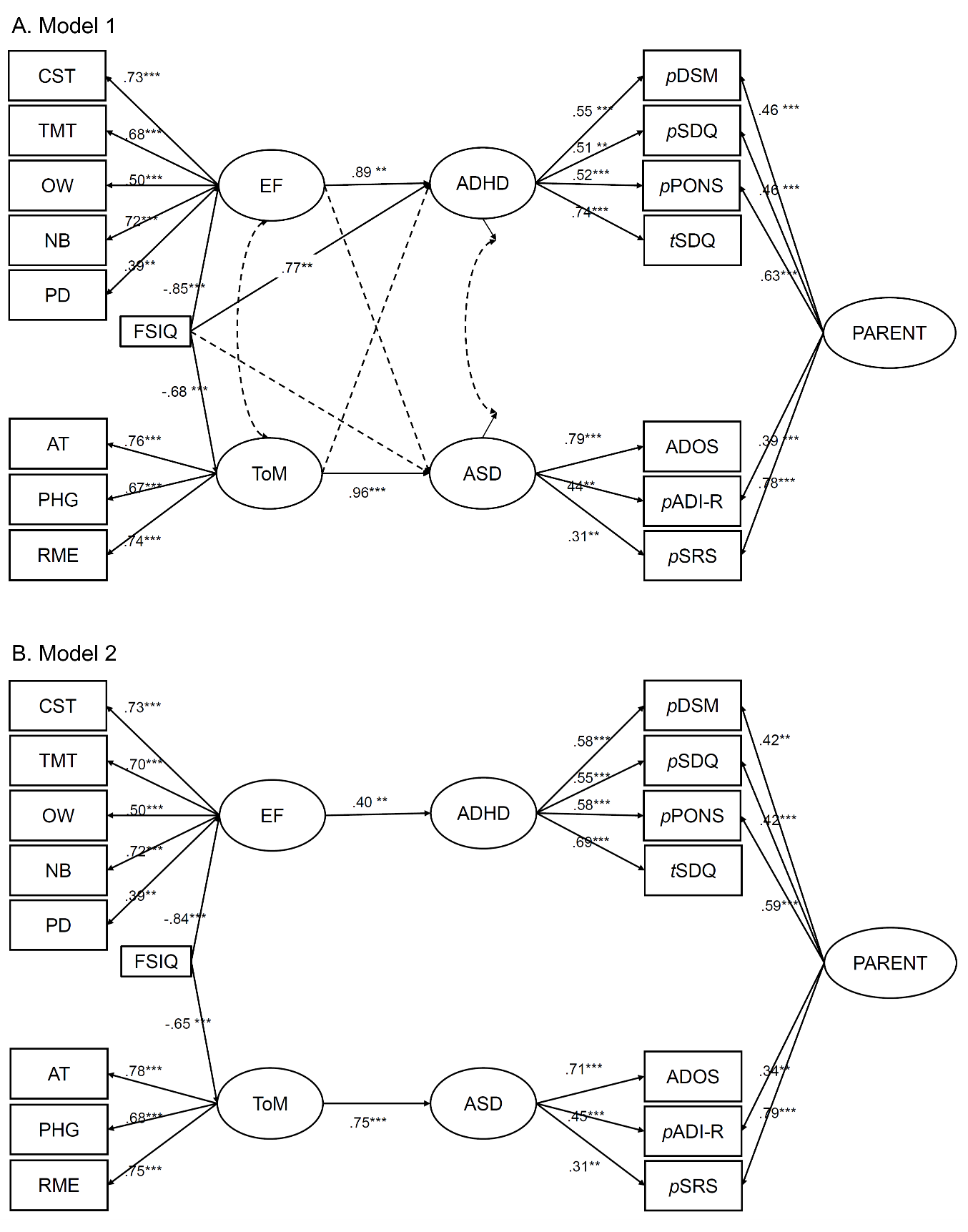 |
| --- |
| Figure S1: Controlling for IQ in the final model. Latent factors EF, ToM, ASD and ADHD were regressed on IQ in the full final model (Figure A), nonsignificant paths were represented by dotted lines. These paths were between EF and ASD (*β* = -.21), ToM and ADHD (*β* =.34), IQ and ASD (*β* =.09), ASD and ADHD (*r* = .018) and between EF and ToM (*r* = .09). The parsimonious model is presented in Fig. S1B. List of abbreviations CST = card sort task, TMT = Trail-Making Test, OW = Opposite Worlds, NB = Number Backward, PD = planning/drawing task, AT = animated triangle, PHG = penny hiding games, RME = Reading the Mind in the Eye tasks, EF = executive function, ToM = theory of mind. Abbreviations for ASD or ADHD measures: ADOS = Autism Diagnostic Observation Schedule, ADI-R = Autism Diagnostic Interview-Revised, SRS = Social Responsiveness Scale, DSM = Diagnostic and Statistical Manual of Mental Disorders*,* SDQ = Strengths and Difficulties Questionnaire, PONS = Profile of Neuropsychiatric Symptoms. The prefix *p* indicates parent-based reports whereas the prefix *t* indicates a teacher-based report. The significant levels * *p* < .05, ** *p* <.01, and *** *p* < .001. |

## Sensitivity analysis 2: A model using data from the 14-16 year-old children

The model we have constructed so far presumed the persistence of ASD and ADHD symptoms over the ages of 10-16 years extending over two waves of investigation. To investigate whether the model stands without the presumption, a sensitivity analysis was undertaken using data collected from the children when they were 14-16 years, removing measure indices collected at Wave 1 investigation. Fit was excellent for the model from the adolescent data (χ^2^[46] = 46.3, *p* = .46; CFI >.99; TLI >.99; RMSEA = .008; AIC = 3918.9; BIC = 4028.3; Model 3 Figure S2A). Removing nonsignificant paths between EF and observed ASD symptoms (*β* = .06, *p* = .81), ToM and ADHD (*β* = .25, *p* = .17), EF and ToM (*r* = .06, *p* = .76), and paths from IQ to ADHD (*β* = .42, *p* = .09) and ASD symptoms (*β* = -.11, *p* = .64) resulted in a parsimonious model with a fit not significantly different from the initial model (Sattora-Bentler scaled Δχ^2^[5] = 6.51, *p* = .26; CFI =.99; TLI =.99; RMSEA = .018; Figure S2B). The specific relations between EF and ADHD (*β* = .38, *p* = .005) and between ToM and observed ASD symptoms (*β* = .39 *p* < .001) were retained, controlling for the association between IQ and impairments in EF (*β* = -.84, *p* < .001) and ToM (*β* = -.72, *p* < .001). The model fit held even after excluding six children whose testing days were separated by more than the 2×SD days of the mean (χ^2^[51] = 47.7, *p* = .61; CFI >.99; TLI >.99; RMSEA < .001; AIC = 3683.6; BIC = 3777.7) with significant associations between EF and ADHD (*β* = .69, *p* = .01), ToM and ASD symptoms (*β* = .33, *p* = .02); and non-significant associations between EF and ASD (*β* = .003, *p* = .99), ToM and ADHD (*β* = .29, *p* = .10), ASD and IQ (*β* = .29, *p* = .10) and ADHD and IQ (*β* = .47, *p* = .08), even after controlling for the associations between IQ and impairments in EF (*β* = -.84, *p* < .001) and ToM (*β* = -.70, *p* < .001). The parsimonious model, which did not differ in fit from the initial model (Sattora-Bentler scaled Δχ^2^[5] = 5.90, *p* = .30; CFI >.99; TLI >.99; RMSEA < .001) consisted of the associations between EF and ADHD (*β* = .38, *p* < .001), ToM and ASD (*β* = .40, *p* < .001) and ASD and ADHD symptoms (*r* = .51, *p* < .001), with the associations between IQ and impairments in EF (*β* = -.83, *p* < .001) and ToM (*β* = -.72, *p* < .001) controlled for.

| 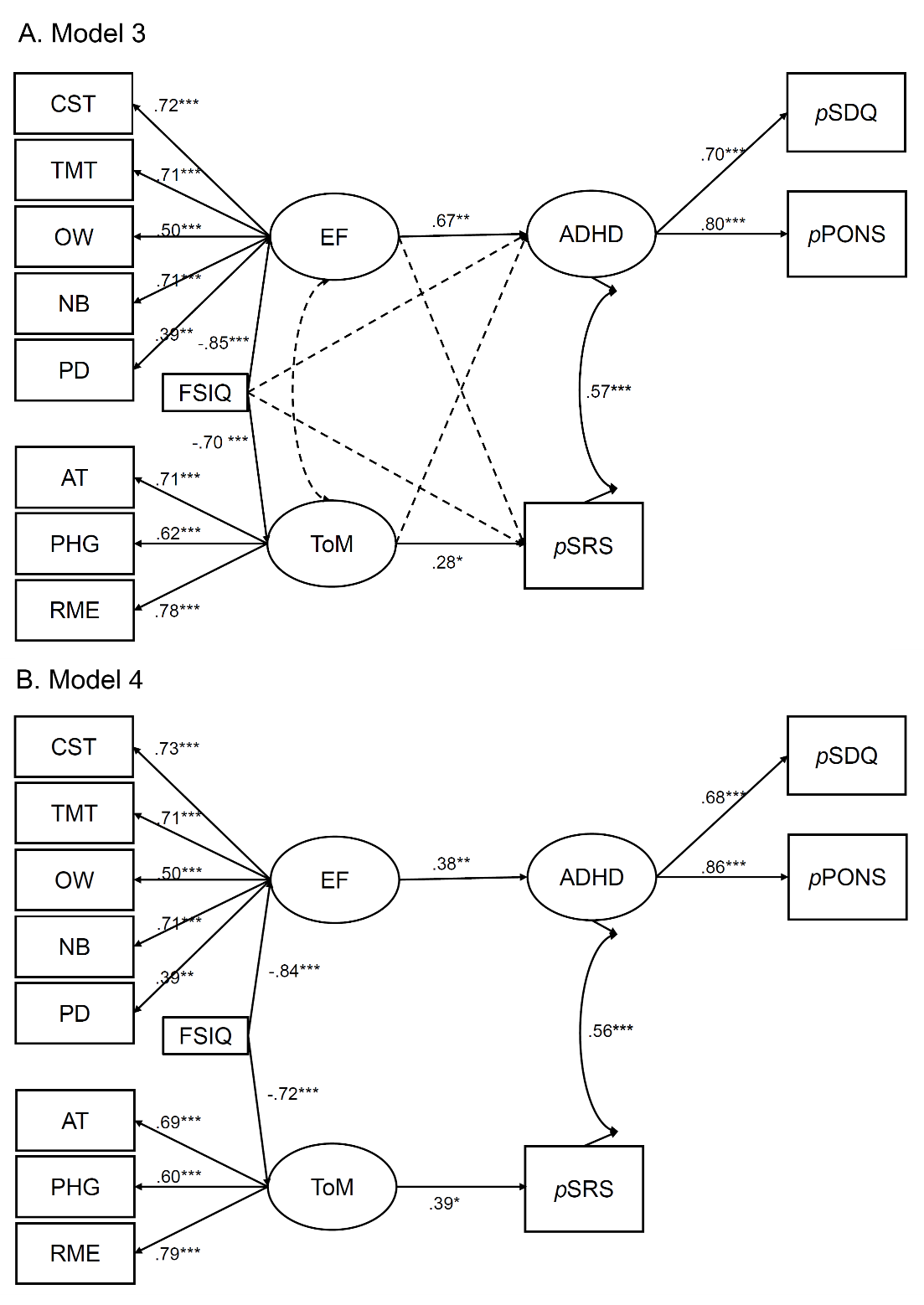 |
| --- |
| Figure S2: Model including only measures from collected from the adolescents at the age of 14-16 years old**.** Latent factors EF, ToM and ADHD and observed ASD symptoms indexed using the parent SRS (pSRS) measures were regressed on IQ (Figure S2A). The nonsignificant paths were between EF and pSRS (*β* = .06), ToM and ADHD (*β* =.25), IQ and pSRS (*β* =.11), and IQ and ADHD (*β* =.42). The parsimonious model is presented in Figure B. List of abbreviations CST = card sort task, TMT = Trail-Making Test, OW = Opposite Worlds, NB = Number Backward, PD = planning/drawing task, AT = animated triangle, PHG = penny hiding games, RME = Reading the Mind in the Eye tasks, EF = executive function, ToM = theory of mind. Abbreviations for ASD or ADHD measures: ADOS = Autism Diagnostic Observation Schedule, ADI-R = Autism Diagnostic Interview-Revised, SRS = Social Responsiveness Scale, DSM = Diagnostic and Statistical Manual of Mental Disorders*,* SDQ = Strengths and Difficulties Questionnaire, PONS = Profile of Neuropsychiatric Symptoms. The prefix *p* indicates parent-based reports whereas the prefix *t* indicates a teacher-based report. The significant levels * *p* < .05, ** *p* <.01, and *** *p* < .001. |

# Supplement References

1. Santosh PJ, Gringras P, Baird G, Fiori F, Sala R. Development and psychometric properties of the parent version of the Profile of Neuropsychiatric Symptoms (PONS) in children and adolescents. BMC Pediatr. 2015; 15:62.

2. Santosh PJ, Baird G, Pityaratstian N, Tavare E, Gringras P. Impact of comorbid autism spectrum disorders on stimulant response in children with attention deficit hyperactivity disorder: a retrospective and prospective effectiveness study. Child Care Health Dev. 2006; 32:575-583.

3. Tregay J, Gilmour J, Charman T. Childhood rituals and executive functions. Br J Dev Psychol. 2009; 27:283-296.

4. Grant DA, Berg EA. A behavioral analysis of degree of reinforcement and ease of shifting to new responses in a Weigl-type card-sorting problem. J Exp Psychol. 1948; 38:404-411.

5. Luria AR, Pribram KH, Homskaya ED. An experimental analysis of the behavioral disturbance produced by a left frontal arachnoidal endothelioma (meningioma). Neuropsychologia. 1964; 2:257-280.

6. Reitan RM. Validity of the trail making test as an indicator of organic brain damage. Percept Mot Skills. 1958; 8:271.

7. Booth R, Charlton R, Hughes C, Happé F. Disentangling weak coherence and executive dysfunction: planning drawing in autism and attention-deficit/hyperactivity disorder. Philos Trans R Soc Lond B Biol Sci. 2003; 358:387-392.

8. Manly T, Anderson V, Nimmo-Smith I, Turner A, Watson P, Robertson IH. The differential assessment of children's attention: the Test of Everyday Attention for Children (TEA-Ch), normative sample and ADHD performance. J Child Psychol Psychiatry. 2001; 42:1065-1081.

9. Cohen M. Children’s memory scale. San Antonio, TX: The Psychological Corporation; 1997.

10. Baron-Cohen S, Wheelwright S, Scahill V, Lawson J, Spong A. Are intuitive physics and intuitive psychology independent? A test with children with Asperger syndrome. J Dev Learn Disord. 2001; 5:47-78.

11. Baron-Cohen S. Out of sight or out of mind? Another look at deception in autism. J Child Psychol Psychiatry. 1992; 33:1141-1155.

12. Happé FG. An advanced test of theory of mind: understanding of story characters' thoughts and feelings by able autistic, mentally handicapped, and normal children and adults. J Autism Dev Disord. 1994; 24:129-154.

13. Castelli F, Frith C, Happé F, Frith U. Autism, Asperger syndrome and brain mechanisms for the attribution of mental states to animated shapes. Brain. 2002; 125:1839-1849.

14. Jones CR, Pickles A, Falcaro M, Marsden AJS, Happé F, Scott SK, Sauter D, Tregay J, Phillips RJ, Baird G, et al. A multimodal approach to emotion recognition ability in autism spectrum disorders. J Child Psychol Psychiatry. 2011; 52:275-285.

15. Hughes C, Adlam A, Happe F, Jackson J, Taylor A, Caspi A. Good test--retest reliability for standard and advanced false-belief tasks across a wide range of abilities. J Child Psychol Psychiatry. 2000; 41, 483-90

16. Sullivan K, Zaitchik D, Tager-Flusberg H. Preschoolers can attribute second-order beliefs. Dev Psychol. 1994; 30, 395-402

17. Bowler DM. “Theory of Mind” in Asperger's syndrome. J Child Psychol Psychiatry. 1992; 33:877-893.
